# Upregulated Expression of Intestinal Antimicrobial Peptide HD5 Associated with Renal Function in IgA Nephropathy

**DOI:** 10.1155/2020/2078279

**Published:** 2020-02-05

**Authors:** Shaozhen Feng, Zhong Zhong, Jinjin Fan, Xiaoyan Li, Dianchun Shi, Lanping Jiang

**Affiliations:** ^1^Department of Nephrology, The First Affiliated Hospital Sun Yat-sen University, Guangzhou, Guangdong, China; ^2^Key Laboratory of Nephrology, Guangdong Province, Guangzhou, Guangdong, China

## Abstract

**Purpose:**

It was reported that gut-kidney axis may play an important role in IgA nephropathy (IgAN). Previous five GWASs of different populations for IgAN have discovered several genes related to intestinal immunity, including *DEFA* gene. However, the roles of the encoded proteins of *DEFA5*/*6* which were called intestinal antimicrobial peptides HD5 and HD6 were not clear in kidney disease, such as IgAN. The purpose of this study was to clarify the association of HD5 and HD6 with IgAN.

**Methods:**

We measured HD5 and HD6 in serum, urine, and kidney of IgAN patients and normal controls by ELISA, Western blot, and immunofluorescence. The association of HD5 or HD6 levels with clinical and pathologic phenotypes was analyzed.

**Results:**

Serum levels of HD5 and HD6 were significantly higher in IgAN patients than those in normal controls. Baseline serum HD5 levels were significantly associated with eGFR (*P* = 0.002) and tubular atrophy/interstitial fibrosis (*P* = 0.002) and tubular atrophy/interstitial fibrosis (*P* = 0.002) and tubular atrophy/interstitial fibrosis (*P* = 0.002) and tubular atrophy/interstitial fibrosis (

**Conclusions:**

In IgAN patients, an elevated serum HD5 level at the time of renal biopsy was associated with poor renal outcomes. HD5 rather than HD6 was probably associated with renal function of IgAN patients.

## 1. Introduction

IgA nephropathy (IgAN) is the most common primary glomerulonephritis among individuals undergoing renal biopsy [1, 2]. It is characterized by the deposition of IgA1 immune complexes in the mesangial area of glomeruli [3], and its deposition induced various histopathological lesions including mesangial cell proliferation and accumulation of the extracellular matrix [4, 5].

The precise mechanism of IgAN is not completely understood. Genetic risk factors, epigenetics, environmental factors, and mucosal immunity are thought to play important roles in the pathogenesis of IgAN [6, 7]. It is reported that the gut-kidney axis is active in IgAN, and intestinal mucosal immunity modified by genetics, gut microbiome, and diet may probably be involved in the development of IgAN [8, 9]. Five large genome-wide association studies (GWAS) of IgAN patients, including our previous studies of Han Chinese IgAN patients, discovered several genes related to intestinal immunity, including *DEFA*, *ITGAM-ITGAX*, *CARD9*, *VAV3*, and *TNFSF13* [10–14]. A genome-wide linkage scan of the Chinese IgAN family also revealed that the *DEFA* region was significantly associated with the susceptibility to IgAN [15]. Further studies of *DEFA* polymorphism and copy number of *DEFA1/A3* in Chinese population indicated that *DEFA* variants were strongly associated with IgAN [16, 17]. However, the roles of encoded proteins of *DEFA* (*α*-defensins), especially two enteric *α*-defensins in the pathogenesis of IgAN, were still not well understood.

Six types of *α*-defensins have been identified in human, including human neutrophil peptides (HNP) 1-4 (encoded by *DEFA*1/3 and *DEFA*4) and human defensins (HD) 5 and 6 (encoded by *DEFA*5 and *DEFA*6). HNPs are normally localized in the granules of neutrophils [18–20]. HD5 and HD6, the most abundant antimicrobial peptides in the small intestine, are constitutively expressed in Paneth cells and induced to secretion by bacterial, cholinergic, or other stimuli [21–26], which contribute to host defense against enteric pathogens [27, 28]. Mouse Paneth cell *α*-defensins have potent selective microbicidal activities against pathogenic bacteria but minimal or no bactericidal activity against commensal bacteria [29]. Furthermore, HD5, which could induce intestinal epithelial cells to increase IL-8 secretion, may also act as a regulator of the intestinal inflammatory response [30]. Besides, in Paneth cells, HD5 are also identified in epithelial cells of the small intestine [31], female reproductive tract [32], nasal organ, and bronchus [33], which suggests that HD5 probably act as an effector of innate immunity.

Alpha-defensins are immunomodulatory peptides and show to be modulated in various disease states [34]. However, until now, with the exception of pyelonephritis [35], no study of kidney diseases focuses on intestinal antimicrobial peptides HD5 and HD6. Whether expressions of HD5 and HD6 are altered and what are the potential roles of them in IgAN are not clear. In the present study, we aimed to investigate the expression patterns of HD5 and HD6 in the serum, urine, and renal tissue of patients with IgAN and to study the association with clinical-pathological characteristics.

## 2. Materials and Methods

### 2.1. Patients and Normal Control Subjects

The study was carried out in accordance with principles of the Institutional Review Board of the First Affiliated Hospital of Sun Yat-sen University. All participants signed informed consent forms. Fifty-three Chinese patients with primary IgAN confirmed by renal biopsy were studied. Exclusion criteria included diabetes, SLE, Henoch-Schonlein purpura, hepatic disease, and the treatment with corticosteroids and immunosuppressive drugs during the previous 3 months. IgAN patients were enrolled from July 2012 to February 2013 and followed up till the end of August 2018 with recording clinical and pathological parameters including serum creatinine, eGFR, and urinary protein. As pathological parameters, mesangial hypercellularity (M), endocapillary hypercellularity (E), segmental glomerulosclerosis (S), and tubular atrophy/interstitial fibrosis (T) were estimated according to the definition of the Oxford classification. Thirty-five age- and sex-matched healthy donors without microscopic hematuria and proteinuria were recruited as normal controls. Blood samples and first-morning urine samples of IgAN patients were obtained at the day of renal biopsy. Serum and urine were isolated via centrifuge and stored at a -80°C refrigerator.

### 2.2. Measurement of Serum and Urine Levels of HD5 and HD6

Serum and urine levels of HD5 and HD6 were measured by the human HD5 and HD6 ELISA kit (USCN Life Science, Wuhan, China) according to the manufacturer's instructions [36]. Serum was diluted 1 : 10 in the appropriate buffer and transferred to microtitre plates for incubation procedures. Urine was undiluted. Duplicate determination was carried out. Absorbance was measured at 450 nm using the SpectraMax Plus 384 microplate reader (Molecular devices, CA, USA). The concentrations of *α*-defensins were calculated according to the standard curve.

### 2.3. Western Blot

Western blot was used to confirm the result of serum HD5 measurement by ELISA. Firstly, 1 mL serum of each sample was centrifuged with the Amicon Ultracentrifuge 30K filter to remove proteins such as albumin. Secondly, the filtrate serum samples were proceed to freeze-drying and resolved in 30 *μ*L PBS buffer, which were used to electrophoresis on 4%-20% gradient polyacrylamide gels and then transferred to the PVDF membrane. The blots were incubated overnight at 4°C with the rabbit anti-HD5 polyclonal antibody (Zen-Bio). A peroxidase-conjugated goat anti-rabbit antibody (Amersham Pharmacia Biotech) was used as the secondary antibody. The membranes were developed using the enhanced ECL detection system (Thermo Fisher).

### 2.4. Immunofluorescence Analysis of Tissue Sections

Renal biopsies of three IgAN patients and two kidney donors used as normal kidney tissues were formalin-fixed and subsequently paraffin-embedded. Following serial 4 *μ*m sections were deparaffinized and rehydrated; antigen retrieval was performed in 0.01 mol/L sodium citrate buffer, pH 6.0, in an antigen retriever (121°C) for 15 minutes. After blocking, tissue sections were individually incubated with the mouse anti-HD5 antibody (Abcam, Cambridge, USA) and mouse anti-HD6 antibody (Abcam) overnight at 4°C. Double-labeled immunofluorescence was performed to help localize HD5 and HD6 expression in the kidney using the rabbit monoclonal antibody against aquaporin-1 (AQP-1; Abcam) which labels the proximal tubules. After washing, bound antibodies were detected by the Alexa 488-conjugated goat anti-mouse or anti-rabbit IgG secondary antibody (Molecular Probes, OR, USA). Nuclei were stained with DAPI (Sigma, St. Louis, USA), and slides were mounted with the ProLong Gold Antifade Reagent (Molecular Probes). Evaluation was performed and analyzed with an LSM 510 Meta confocal laser scanning microscope (Carl Zeiss, Jena, Germany).

### 2.5. Statistical Analysis

To calculate statistically significant differences between groups, samples were analyzed by Student's *t*-test for values with normal distribution and by the Mann-Whitney test for values without normal distribution. Relationships between the serum HD5/HD6 level and clinical parameters were calculated using Spearman's rank correlation and multiple linear regression analysis. Values with nonnormal distribution were log converted in the multivariate linear regression analysis. Renal survival rates in IgAN patients were assessed by the Kaplan-Meier method. The endpoint of renal survival was eGFR < 30%. Differences in the survival curves of the high serum HD5 level and low HD5 level groups were analyzed by the log-rank test. A Cox regression model was used to estimate the size of the effect, choosing a forward stepwise procedure to assess the impact of multiple covariates for renal prognosis. The results of multivariable analysis were expressed by a hazard ratio (HR) and 95% confidence intervals. Results are presented as mean ± SD or median (25^th^ and 75^th^). *P* < 0.05 was considered statistically significant in all tests. Data analyses were performed using GraphPad Prism version 4 and SPSS 16.0 software.

## 3. Results

### 3.1. Elevated Serum Levels of HD5 and HD6 in Chinese IgAN Patients

**Figure 1:**
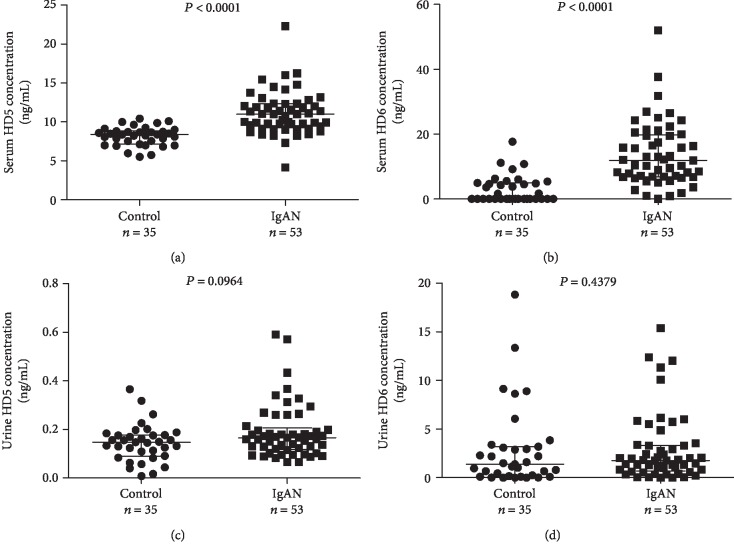
Scatter dot plots of HD5 and HD6 measurements in IgAN patients. ELISA was used to determine the serum levels of HD5 (a) and HD6 (b), as well as urinary levels of HD5 (c) and HD6 (d) in Chinese IgAN patients and normal controls. Line indicates median with interquartile range. *P* value analysis: nonparametric test (Mann-Whitney *U*).

Serum and urine were collected from 53 IgAN patients and 35 age- and sex-matched normal controls (Table 1). The median serum levels of HD5 and HD6 in IgAN patients were significantly higher than those in normal controls (10.87 (9.08, 12.23) ng/mL versus 8.36 (7.12, 8.80) ng/mL for HD5; 11.91 (6.85, 19.71) ng/mL versus 0.01 (0.00, 4.99) ng/mL for HD6) (Figures 1(a) and 1(b)). However, there was no statistical difference of urinary levels of HD5 and HD6 between IgAN patients and normal controls (0.17 (0.12, 0.21) ng/mL versus 0.15 (0.09, 0.18) ng/mL for HD5, 1.78 (0.71, 3.35) ng/mL versus 1.40 (0.17, 3.19) ng/mL for HD6) (Figures 1(c) and 1(d)).

### 3.2. Correlations of Serum Levels of HD5 and HD6 with Clinical-Pathological Features

Correlations between the serum levels of HD5/HD6 and clinical-pathological variables were analyzed by the Spearman rank correlation test (Table 2). The serum HD5 level in IgAN patients was positively correlated with age, CKD grade, serum creatinine, 24 hr proteinuria, segmental glomerulosclerosis, and tubular atrophy/interstitial fibrosis in renal pathology, while negatively correlated with eGFR (*P* < 0.05). In stepwise multivariate regression analysis with age, gender, eGFR, log transformed 24 hr proteinuria, segmental glomeruloscierosis, tubular atrophy/interstitial fibrosis, and log transformed CRP (C-reactive protein), only eGFR and tubular atrophy/interstitial fibrosis were associated with serum HD5 (total adjusted *R* = 0.806) (Table 3).

**Figure 2:**
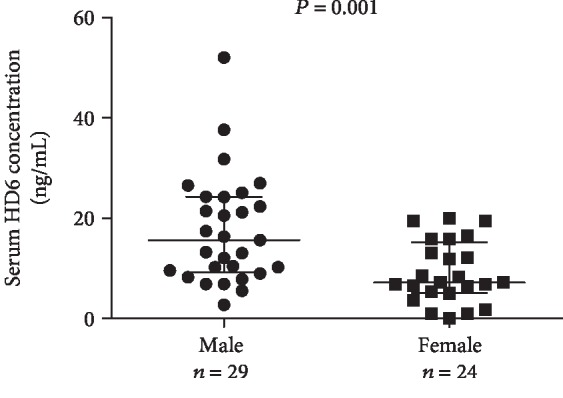
Scatter dot plot and gender difference of serum HD6 in IgAN patients. ELISA was used to determine the serum levels of HD6 in Chinese IgAN patients as Figure 1. Line indicates median with interquartile range. *P* value analysis: nonparametric test (Mann-Whitney *U*).

A serum HD6 level was only correlated with gender (*r* = ‐0.388, *P* = 0.004) (Table 2) and was higher in male than in female patients (Figure 2). Stepwise multivariate regression analysis with the same explanatory variables as HD5 also revealed significant association of HD6 with gender (total adjusted *R* = 0.389) (Table 3). In normal controls, there was no statistical correlation of low serum levels of HD5 and HD6 with clinical parameters ([Supplementary-material supplementary-material-1]).

### 3.3. Comparison of IgAN Patients with a High or Low Serum HD5 Level

Since the median serum HD5 level for IgAN patients at the time of renal biopsy was 10.87 ng/mL (Figure 1(a)), fifty-three IgAN patients were classified according to a high (≥11 ng/mL, *n* = 23) or a low (<11 ng/mL, *n* = 22) serum HD5 level. Both eGFR at the time of renal biopsy and at final follow-up, as well as mesangial hypercellularity, were significantly lower in the group with a high serum HD5 level, rather than the group with a low serum HD5 level (Table 4). Serum creatinine, 24 hr proteinuria levels, and tubular atrophy/interstitial fibrosis at baseline were significantly higher in the group with a high serum HD5 level, but age, CRP at baseline, and drugs for treatment did not differ between the two groups.

### 3.4. Relationship between the Renal Survival and Serum HD5 Level

**Figure 3:**
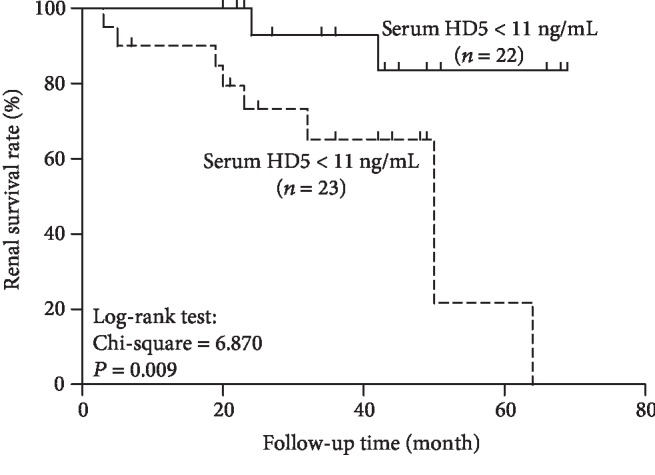
Kaplan-Meier analysis of cumulative renal survival of patients with IgAN based on the serum HD5 level at renal biopsy. Survival curves significantly differ (log-rank test, chi − square test = 0.870, *P* = 0.009).

Total 45 IgAN patients had follow-up data. The mean follow-up period was 34.15 ± 17.34 (3-69) months. The endpoint was a 30% of decline in eGFR from baseline after follow-up. Thirteen (28.89%) patients developed eGFR decline over 30% from baseline including two kidney transplants and two dialysis. We compared the renal survival of patients based on a high (≥11 ng/mL) or a low (<11 ng/mL) serum HD5 level. Ten patients with serum HD5 levels above the median level (≥11 ng/mL) developed eGFR decline over 30% from the baseline. Kaplan-Meier analysis revealed that patients with a high level of serum HD5 at baseline had a significant poor renal outcome (log-rank test, chi − square test = 0.870, *P* = 0.009) (Figure 3).

To correct for bias caused by univariate analysis, baseline serum HD5 as well as other parameters were examined in a multivariate Cox regression analysis (after adjustment for age, gender, baseline eGFR, log transformed 24 hr proteinuria, log transformed CRP, and pathologic finding tubular atrophy/interstitial fibrosis). Baseline serum HD5 was found to be an independent prognostic factor (HR = 1.239, *P* = 0.029) for IgAN patients (Table 5).

### 3.5. Expression Level of HD5 and HD6 in the Renal Tissue of IgAN Patients

**Figure 4:**
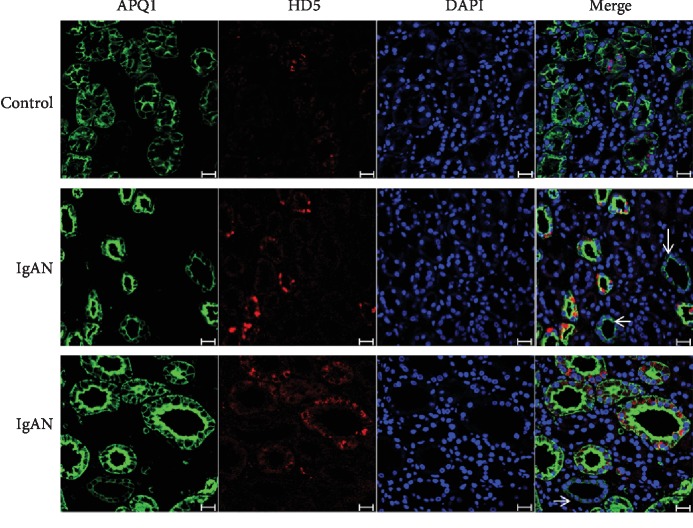
Expression levels of HD5 in the kidney of IgAN. Human kidney labeled for HD5 (red), proximal tubule marker AQP-1 (green), and nuclei (blue). HD5 (red) showed production in the damaged proximal tubules with the exception of normal proximal tubules (arrow). Scale bar = 50 *μ*m.

Immunofluorescence analysis showed that in the renal biopsies of IgAN patients, specific signals of HD5 were double-stained with the proximal tubular marker AQP-1 and increasingly observed in the damaged proximal tubules (Figure 4). The glomeruli and interstitium showed no HD5 expression. Since the mouse anti-HD5 antibody (8C8) recognized the HD5 propeptide (Abcam), it suggested that HD5 was expressed as a propeptide in renal tubules. However, neither renal biopsies of IgAN patients nor normal controls showed HD6 immunoreactivity with the tested HD6 antibody (Fig. [Supplementary-material supplementary-material-1]).

## 4. Discussion

The present study provided an initial investigation of HD5 and HD6 in serum, urine, and renal tissue of IgAN patients, which revealed elevated serum HD5 in IgAN patients and further found the association with eGFR and tubular atrophy/interstitial fibrosis. IgAN patients with serum HD5 above the median level had a significant poor renal outcome. Serum HD5 was an independent prognostic factor of IgAN patients. In addition, we demonstrated abundant expression of HD5 in the damage proximal tubules in the kidney of IgAN patients by immunostaining, while no immunoreactive HD6 was observed in the kidney. Elevated serum HD6 levels were found to be associated with gender in IgAN patients. The current findings suggested that HD5 rather than HD6 may probably play important roles in regulating kidney function of IgAN.

**Figure 5:**
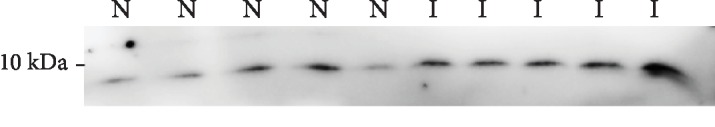
Western blot analysis of serum HD5 in normal controls (N) and IgAN patients (I).

There were some evidences suggesting that HD5 or HD6 persists in serum with very low concentration [36, 38, 39]. We also detected serum HD5 by ELISA and found a relatively low level of HD5 in peripheral blood both from IgAN patients and normal controls. In order to confirm the serum HD5 measured by ELISA, we determined serum samples by Western blot (Figure 5). There were specific immunoreactive bands of HD5 in serum of IgAN patients and normal controls. Serum HD5 concentrations were higher in IgAN patients than those in normal controls which was consistent with the ELISA results. mRNA and protein of HD5 could not be detected in the whole white blood cells of IgAN patients and normal controls using PCR and Western blot (data not shown), which indicated that serum HD5 may not be derived from white blood cells. HD5 are secreted from Paneth cells in response to bacterial stimuli [23, 24]. Upper respiratory tract infection [40–43], intestinal pathogens [44], and periodontal bacteria [45] were reported to be associated with IgAN. Intestinal bacteria could activate the innate immune system and were suggested to possibly trigger and aggravate IgAN [44, 46, 47]. It is hypothesized that antigens from extrinsic microorganisms or recurrent mucosal infection may induce lymphocyte-mediated immune response in IgAN, at the same time stimulate HD5 secretion from Paneth cells in response to phagocytic stimuli. Elevated levels of HD5 in the sera of IgAN patients probably stem from secretion in the small intestine due to stimulation since mature HD5 are small-sized peptides with 32 amino acids which could be exported from the Paneth cells into blood [38, 39]. Moreover, HD5 as a small molecule would be filtrated in glomerulus. Low eGFR can decrease the urine excretion of HD5, resulting in the increase of serum concentration of HD5 in IgAN patients.

If the hypothesis that serum HD5 stem from the small intestine was valid, IgAN patients with an elevated serum HD5 level probably had high secretion of HD5 in the small intestine. HD5, the most abundant antimicrobial peptides in the small intestine, are known to contribute to host defense against enteric pathogens [27]. HD5 exhibits high antimicrobial activity against Enterobacteriaceae. Overexpression of HD5 resulted in increased Clostridiaceae by decreasing competing bacteria families, leading to chronic/relapsing pouchitis [48]. Expression balance of HD5 is essential for the intestinal homeostasis [49]. Several recent reviews mentioned an active gut-kidney axis in chronic kidney disease [8, 9, 50–52]. Intestinal mucosal immunity modified by genetics, microbiota, and diet may probably be involved in the development of IgAN. Clinical-pathological analysis revealed that serum HD5 levels were associated with eGFR in IgAN patients. IgAN patients with elevated serum HD5 above the median level had a worse renal outcome than those with low serum HD5. Furthermore, serum HD5 was an independent risk factor of IgAN progression indicating that serum HD5 may be a clinical marker that could reflect renal functions in IgAN. It was proposed that HD5 may play an indirect pathogenic role in IgAN by modulating intestinal microbiota. HD5 may have a key role as one mechanism linking the enteric microbiota and IgAN.

Elevated serum HD5 levels were also associated with tubular atrophy/interstitial fibrosis in IgAN patients. Moreover, HD5 were found to be expressed in the damaged proximal tubules with the exception of normal proximal tubules in the renal biopsies of IgAN patients by immunostaining. It was reported that in patients with pyelonephritis, HD5 was primarily produced in the distal nephron and collecting tubules and its expression was increased [35]. HD5 was also expressed in epithelial cells of the female reproductive tract [32], nasal organ, and bronchus [33], which suggested that HD5 produced in locations where it will be positioned to have optimal antimicrobial activity. Whether HD5 was just considered as a marker for tubular atrophy/interstitial fibrosis or HD5 indeed has any effect on proximal tubules in IgAN, more experiments *in vitro* cell culture should be further studied.

Our study has certain limitations. First, a small proportion of patients were evaluated, although the current results strongly suggested that serum HD5 was an independent prognostic factor in IgAN patients. Second, the associations of serum HD5 with renal function were only studied in IgAN patients and did not include other CKD patients. Subsequent studies should increase the sample size including IgAN and other CKD patients, and a long-term follow-up study is needed to verify the conclusions of the present study.

## 5. Conclusions

The antimicrobial, antiviral, and immunomodulatory activities of HD5 are host protective during infection [27, 48, 49, 53, 54]. Considering the relationship between the upregulated serum HD5 with clinical-pathological parameters and long-term renal outcome, as well as the expression of HD5 in the damage proximal tubules in IgAN patients, the present study indicated that HD5 as a regulator of intestinal immunology may be a reliable marker of the progression of IgAN and associate with the renal function of IgAN.

## Figures and Tables

**Table 1:** Clinical information of IgAN patients and normal controls at baseline.

	Normal controls (*N* = 35)	IgAN patients (*N* = 53)	*P* value
Gender (male/female)	16/19	29/24	0.414
Age (years)	35.93 ± 9.56	32.76 ± 10.01	0.567
eGFR (mL/min/1.73 m^2^)	114.07 ± 20.18	77.13 ± 38.72	0.006
Serum creatinine (*μ*mol/L)	62.51 (56, 73.5)	99 (69, 152)	<0.001
Proteinuria range (g/24 h)	0^a^	1.18 (0.67, 3.21)	—
WBC (×10^9^/L)	6.49 ± 1.25	7.21 ± 1.79	0.047
Neutrophil (×10^9^/L)	3.56 ± 0.98	4.53 ± 1.66	0.003
Monocyte (×10^9^/L)	0.40 ± 0.14	0.49 ± 0.22	0.032
Lymphocyte (×10^9^/L)	2.36 ± 0.57	1.98 ± 0.55	0.003
C-reactive protein (mg/L)	—	0.83 (0.82, 1.22)	—
IgA (g/L)	—	2.83 ± 0.79	—
IgG (g/L)	—	10.01 ± 3.33	—
IgM (g/L)	—	1.40 ± 1.03	—
C3 (g/L)	—	1.01 ± 0.19	—
C4 (g/L)	—	0.23 ± 0.07	—
*Oxford MEST*		*Number (%)*	
M0	—	14 (26.42%)	—
M1	—	39 (73.58%)	—
E0	—	15 (28.30%)	—
E1	—	38 (71.70%)	—
S0	—	47 (88.68%)	—
S1	—	6 (11.32%)	—
T0	—	35 (66.04%)	—
T1	—	13 (24.53%)	—
T2	—	5 (9.43%)	—

eGFR (estimated glomerular filtration rate) was calculated using the modified MDRD formula as follows: eGFR (mL/min/1.73 m^2^) = 175 × (creatinine, mg/dL)^−1.234^ × (age, years)^−0.179^ × (if female, ×0.79) [37]. ^a^Normal controls without proteinuria were included.

**Table 2:** Univariate correlations of serum HD5 and HD6 with clinical-pathological variables in IgAN patients at baseline.

Serum levels	HD5		HD6	
	*r*	*P*	*r*	*P*
Gender (male/female)	-0.062	0.664	**-0.388**	**0.004**
Age (years)	**0.303**	**0.029**	-0.092	0.517
CKD grade	**0.665**	**<0.001**	-0.028	0.842
Serum creatinine (*μ*mol/L)	**0.649**	**<0.001**	0.020	0.889
eGFR (mL/min/1.73 m^2^)	**-0.729**	**<0.001**	0.101	0.480
C-reactive protein (mg/L)	0.131	0.409	0.187	0.236
WBC (×10^9^/L)	-0.065	0.653	0.283	0.047
Neutrophil (×10^9^/L)	0.001	0.992	0.257	0.072
Monocyte (×10^9^/L)	-0.003	0.984	0.085	0.557
Lymphocyte (×10^9^/L)	-0.166	0.249	0.041	0.776
IgA (g/L)	0.097	0.516	0.149	0.317
IgG (g/L)	-0.225	0.129	0.064	0.669
IgM (g/L)	-0.126	0.399	-0.158	0.288
C3 (g/L)	0.129	0.386	-0.002	0.990
C4 (g/L)	0.104	0.487	-0.057	0.704
Proteinuria (g/24 h)	**0.557**	**<0.001**	-0.126	0.388
Mesangial hypercellularity (M)	-0.299	0.065	-0.036	0.806
Segmental glomerulosclerosis (S)	0.218	0.183	0.018	0.900
Endocapillary hypercellularity (E)	-0.112	0.435	-0.227	0.109
Tubular atrophy/interstitial fibrosis (T)	**0.602**	**<0.001**	-0.069	0.631

Correlation is significant at the 0.05 level (Spearman). *P* value < 0.05 was indicated in bold.

**Table 3:** Forward stepwise multivariate regression analysis for predictors of HD5/HD6 in IgAN patients (*n* = 53).

Dependent	Variable	B ± SE	*t*	*P* value	*R*
HD5	eGFR (mL/min/1.73 m^2^)	‐0.038 ± 0.011	-3.355	0.002	0.806
	Tubular atrophy/interstitial fibrosis (T)	2.410 ± 0.760	3.171	0.004	
HD6	Gender	‐9.053 ± 4.052	-2.234	0.034	0.389

SE: standard error. Model is adjusted for age, gender, eGFR, log transformed 24 hr proteinuria, log transformed CRP, mesangial hypercellularity (M), and tubular atrophy/interstitial fibrosis (T).

**Table 4:** Comparison of the high and low serum HD5 levels in IgAN patients.

	Serum HD5 ≥ 11 ng/mL (*n* = 25)	Serum HD5 < 11 ng/mL (*n* = 28)	*P* value
*Baseline*			
Age (years)	34.40 ± 7.50	29.11 ± 8.73	0.144
Serum creatinine (*μ*mol/L)	134.5 (101.75, 191.0)	76.0 (67.0, 96.0)	<0.001
eGFR (mL/min/1.73 m^2^)	54.51 ± 34.498	100.96 ± 27.204	<0.001
Proteinuria (g/24 h)	2.53 (0.732, 4.717)	0.803 (0.658, 1.742)	0.007
CRP (mg/L)	0.836 (0.824, 2.623)	0.836 (0.824, 1.385)	0.761
Mesangial hypercellularity			0.017
M0	11 (44%)	3 (10.71%)	
M1	14 (56%)	25 (89.29%)	
Segmental glomerulosclerosis			0.648
S0	22 (88%)	25 (89.29%)	
S1	3 (12%)	3 (10.71%)	
Endocapillary hypercellularity			0.957
E0	6 (24%)	9 (32.14%)	
E1	19 (76%)	19 (67.86%)	
Tubular atrophy/interstitial fibrosis			0.023
T0	11 (44%)	24 (85.71%)	
T1	9 (36%)	4 (14.29%)	
T2	5 (20%)	0 (0%)	
*Treatment*			
ARB and/or ACE-I	20 (80%)	23 (82.14%)	0.621
Corticosteroid	7 (28%)	8 (28.57%)	0.897
Immunosuppressive drug	1 (4%)	1 (3.57%)	0.956
*Final follow-up*	*n* = 23	*n* = 22	
Observation period (months)	30.30 ± 16.94	39.21 ± 16.25	0.102
Serum creatinine (*μ*mol/L)	145 (114, 307.75)	78 (65, 110)	<0.001
eGFR (mL/min/1.73 m^2^)	48.03 ± 32.79	92.25 ± 33.12	<0.001

All data are mean ± SD or number of patients (%, ratio of group). Two groups are assessed by the *t*-test, Mann-Whitney test, or chi-square test.

**Table 5:** Multivariate Cox regression analysis for renal prognosis according to serum HD5.

	HR (95% CI)	*P* value
Unadjusted	1.373 (1.090, 1.730)	0.007
Adjusted		
Model 1	1.247 (1.070, 1.455)	0.005
Model 2	1.242 (1.062, 1.453)	0.007
Model 3	1.239 (1.022, 1.503)	0.029

Abbreviation: CI: confidence interval. Model 1 was adjusted for age and gender. Model 2 was adjusted as Model 1 plus baseline eGFR log transformed proteinuria (24 hr) and tubular atrophy/interstitial fibrosis. Model 3 was adjusted as Model 2 plus log transformed CRP.

## Data Availability

The data used to support the findings of this study are available from the corresponding author upon request.

## Supplementary Materials

Supplementary Materials Table S1: univariate correlations of serum HD5 and HD6 with clinical variables in normal controls at baseline (*n* = 35). Figure S1: expression levels of HD6 in the kidney. Human kidney labeled for HD6 (red), proximal tubule marker AQP-1 (green), and nuclei (blue). Immunofluorescence staining with the tested HD6 antibody did not display any specific signal in the renal biopsies of IgAN patients and normal donors. Scale bar = 50 *μ*m.
