# Corrigendum to “Upregulated Expression of Intestinal Antimicrobial Peptide HD5 Associated with Renal Function in IgA Nephropathy”

**DOI:** 10.1155/2022/9797825

**Published:** 2022-08-17

**Authors:** Shaozhen Feng, Zhong Zhong, Jinjin Fan, Xiaoyan Li, Dianchun Shi, Lanping Jiang

**Affiliations:** ^1^Department of Nephrology, The First Affiliated Hospital Sun Yat-sen University, Guangzhou, Guangdong, China; ^2^Key Laboratory of Nephrology, Guangdong Province, Guangzhou, Guangdong, China

In the article titled “Upregulated Expression of Intestinal Antimicrobial Peptide HD5 Associated with Renal Function in IgA Nephropathy” [1], the authors identified an error in Figure 3. In Figure 3, the term “HD5 < 11 ng/mL, *n* = 23” should read as “HD5 ≥ 11 ng/mL, *n* = 23.” The correction does not affect the results or conclusions.

The figure should be read as follows:

## Figures and Tables

**Figure 1:**
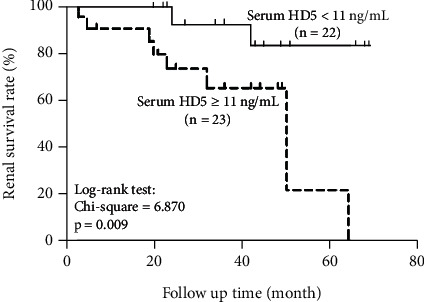
Kaplan-Meier analysis of cumulative renal survival of patients with IgAN based on the serum HD5 level at renal biopsy. Survival curves significantly differ (log-rank test, chi-square test = 0.870, *P* = 0.009).
