# Supplementary material for: Upregulated Expression of Intestinal Antimicrobial Peptide HD5 Associated with Renal Function in IgA Nephropathy
Source: Dis Markers. 2020 Feb 5;2020:2078279. doi: 10.1155/2020/2078279 (PMC7025039; doi:10.1155/2020/2078279)
Supplement: Supplementary Materials — Table S1: univariate correlations of serum HD5 and HD6 with clinical variables in normal controls at baseline (n = 35). Figure S1: expression levels of HD6 in the kidney. Human kidney labeled for HD6 (red), proximal tubule marker AQP-1 (green), and nuclei (blue). Immunofluorescence staining with the tested HD6 antibody did not display any specific signal in the renal biopsies of IgAN patients and normal donors. Scale bar = 50 μm. [file 2078279.f1.pdf]

Supplementary Tables

Table S1 Univariate correlations of serum HD5 and HD6 with clinical variables in normal controls at baseline (n=35)

Serum level	HD5		HD6	
	r	*P*	r	*P*
Gender (male/female)	-0.119	0.495	-0.101	0.565
Age (years)	-0.032	0.858	-0.210	0.234
Serum creatinine (umol/L)	0.003	0.988	0.050	0.779
eGFR (ml/min/1.72 m^2)	0.200	0.256	0.043	0.808
WBC ($*10^9$/L)	0.184	0.330	0.239	0.199
Neutrophil ($*10^9$/L)	0.271	0.122	0.243	0.202
Monocytes ($*10^9$/L)	0.081	0.446	0.208	0.237
Lymphocytes ($*10^9$/L)	-0.026	0.883	0.169	0.339

Correlation is significant at the 0.05 level (Spearman). *P* value < 0.05 was indicated bold.

Supplementary Figures

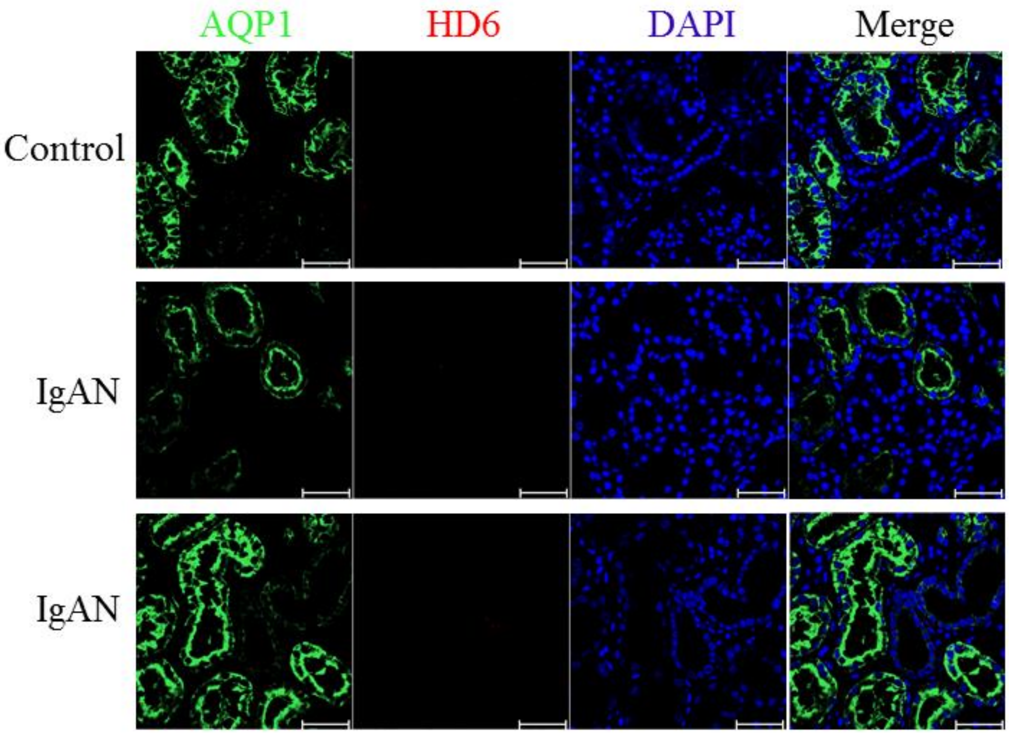

Fig. S1. Expression levels of HD6 in the kidney. Human kidney labeled for HD6 (red), proximal tubule marker AQP-1 (green) and nuclei (blue). Immunofluorescence staining with the tested HD6 antibody did not display any specific signal in the renal biopsies of IgAN patients and normal donors. Scale bar = 50 μm.
